# Changes in Chronotype after Stroke: A Pilot Study

**DOI:** 10.3389/fneur.2014.00287

**Published:** 2015-01-12

**Authors:** Thomas Kantermann, Andreas Meisel, Katharina Fitzthum, Thomas Penzel, Ingo Fietze, Lena Ulm

**Affiliations:** ^1^Chronobiology Unit, Groningen Institute for Evolutionary Life Sciences, University of Groningen, Groningen, The Netherlands; ^2^Clinical Centre, Institute for Occupational, Social and Environmental Medicine, Ludwig-Maximilians University Munich, Munich, Germany; ^3^NeuroCure Clinical Research Center, Charité – Universitätsmedizin Berlin, Berlin, Germany; ^4^Department of Neurology, Center for Stroke Research Berlin, Charité – Universitätsmedizin Berlin, Berlin, Germany; ^5^Center of Sleep Medicine, Charité – Universitätsmedizin Berlin, Berlin, Germany; ^6^Centre for Clinical Research, The University of Queensland, Herston, QLD, Australia

**Keywords:** chronotype, internal time, sleep, stroke, stroke location, NIHSS, mRS

## Abstract

This study aimed to elucidate associations between stroke onset and severity as well as chronotype (phase of entrainment) and internal time of stroke. Fifty-six first-ever ischemic stroke patients participated in a cross-sectional study assessing chronotype (mid-sleep on work-free days corrected for sleep deficit on workdays; MSF_sc_) by applying the Munich ChronoType Questionnaire (MCTQ). The MCTQ was completed twice, on average 68 ± 24 (SD) days post stroke and retrospectively for the time before stroke. To assess the impact of stroke in relation to internal time, InT_stroke_ was calculated as MSF_sc_ minus local time of stroke. Stroke severity was assessed via the standard clinical National Institute Health Stroke Scale (NIHSS) and modified Ranking Scale (mRS), both at hospital admission and discharge. Overall, most strokes occurred between noon and midnight. There was no significant association between MSF_sc_ and stroke onset. MSF_sc_ changed significantly after stroke, especially in patients with more severe strokes. Changes in MSF_sc_ varied with InT_stroke_ – the earlier the internal time of a stroke relative to MSF_sc-before-stroke_, the more MSF_sc_ advanced after stroke. In addition, we provide first evidence that MSF_sc_ changes varied between stroke locations. Larger trials are needed to confirm these findings.

## Introduction

The onset of vascular events exhibits a circadian (about 24-h) pattern, with a prominent peak in the morning hours and a second, smaller peak in the early evening (1–4). This phenomenon is thought being caused by diurnal variations in underlying pathophysiological mechanisms, e.g., platelet aggregation, blood pressure, and catecholamine concentrations (3, 5). Several animal studies demonstrated that cerebrovascular events, in turn, also impact circadian rhythms and sleep architecture (6–9). Longer latencies to fall asleep, fragmented sleep, NREM sleep instability, and hypersomnia have also been described in stroke patients, indicating changes in sleep homeostasis and circadian rhythmicity after stroke (10, 11). Two studies with small sample sizes provided some evidence of altered timing in urinary melatonin as a marker of disturbed circadian rhythms in stroke patients (12, 13). However, knowledge on the interplay between sleep homeostasis, the circadian timing of sleep, and vascular events is still incomplete.

The circadian timing of sleep is regulated by an internal clock, which is synchronized (entrained) by light to the 24-h day (14, 15). The relation between external (local) and internal (circadian) time is called phase of entrainment (16) and people that differ in this trait are referred to as different chronotypes. Chronotype can easily be assessed with the Munich ChronoType Questionnaire [MCTQ; (16)] as the mid-point of sleep on work-free days (MSF), corrected for sleep deficit accumulated across the workweek (MSF sleep corrected; MSF_sc_). MSF_sc_ as a measure for internal time allows calculating the internal time point of a physiological event (17, 18). Social jetlag (the difference between MSF and mid-sleep on workdays, MSW) is a surrogate measure for circadian rhythm disruption and has been linked to elevated heart rate in shift-workers (19), smoking (20), and depression (21). To date, no study has assessed phase of entrainment (chronotype) and social jetlag before and after stroke to compare the impact of stroke in relation to internal time.

The current study was the first to investigate associations between stroke onset and severity and internal time before and after stroke. We hypothesized that (a) there is a correlation between chronotype (MSF_sc_) and stroke onset, (b) experiencing a stroke results in chronotype changes, and (c) the impact of stroke varies with the internal time point a stroke happened.

## Materials and Methods

Between October 2011 and March 2012, 197 consecutive patients treated at the Charité University Hospital Berlin, Germany, who had suffered from first-ever ischemic stroke in the previous 3 months were invited by mail to participate in a cross-sectional assessment of sleep timing before and after stroke. The MCTQ (16) was applied to calculate each patients’ chronotype [MSF_sc_, corrected for sleep deficit on workdays, as MSF minus (sleep duration on work-free days minus average sleep duration of the week)/2; (16)] and social jetlag [difference between MSW and MSF; (20)]. Internal time point of a stroke (Int_stroke_) was calculated as hours since the clock time for the mid-point of sleep on days off corrected for sleep deficit on working days, from MCTQ entries for the time before stroke. The MCTQ was completed twice, on the same day using the same instructions, and on average 68 ± 24 (SD) days post stroke and retrospectively for the time before stroke. Changes in chronotype were assessed as MSF_sc-after-stroke_ minus MSF_sc-before-stroke_.

Clinical information including the time point of stroke was obtained through case report forms, discharge letters, and radiological reports. For patients who were well at bedtime but woke up with symptoms, stroke onset was defined as the time point of awakening (5). Information on stroke severity was collected via the National Institute Health Stroke Scale (NIHSS) (22) and modified ranking scale (mRS) (23, 24), both at hospital admission and discharge. The NIHSS quantifies stroke-related neurological deficits including key aspects of neurological examination (e.g., motor strength, sensation, or language function) (22, 25) and provides a severity score ranging from 0 (indicating no symptoms) to 42. The mRS measures functional outcomes after stroke and consists of 5 grades with 0 = no symptoms, 1 = no significant disability, despite symptoms, 2 = slight disability, 3 = moderate disability, 4 = moderately severe disability, and 5 = severe disability (24, 26). Stroke severity was categorized (NIHSS scores at hospital admission) as mild (≤ 5; 68.6%), moderate (6 to 13; 25.7%), and severe (≥ 14; 5.7%) (27). To assess the effects of stroke location on MSF_sc_, participants were split into two groups with (i) strokes of the anterior circulation providing blood to the cerebrum (middle, anterior and posterior cerebral artery and anterior choroidal artery) and (ii) strokes of the posterior circulation proving blood to the cerebellum and brain stem (ventrobasilar and vertebrobasilar artery). Patients did not receive financial compensation for study participation. As an incentive to participate, patients were offered an appointment at the Center of Sleep Medicine at the Charité University Hospital for an individual sleep evaluation (not part of this study). Written informed consent was obtained from each patient. The ethics committee of the Charité University Berlin approved the study protocol (EA1/214/11).

Data analysis was performed using IBM SPSS Statistics 20.0 for Macintosh. Parametric and non-parametric statistical analyses were performed as appropriate after subjecting the data to the Shapiro–Wilk test to verify if the data were normally distributed. All demographic variables were expressed as mean ± SD. All *p* values were two-tailed and statistical significance was set at a value <0.05 applying Bonferroni correction for multiple testing.

## Results

**Figure 1:**
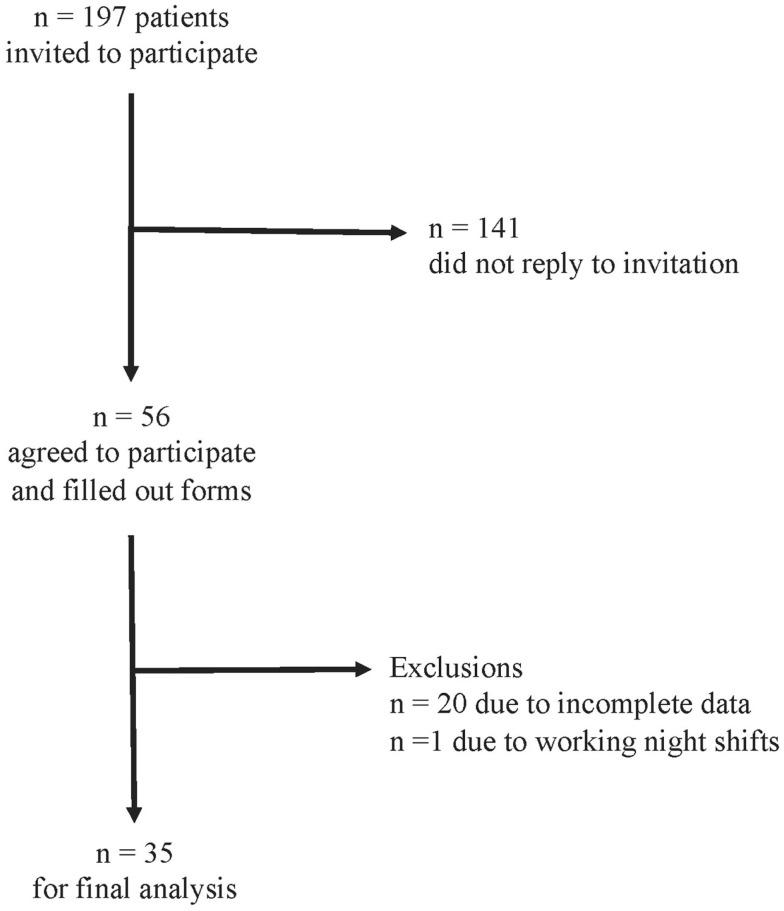
**Study flow chart of patient enrollment and data analysis**.

The response rate was 28% (56 patients). We excluded 20 patients due to incomplete data and one patient due to reporting to work night shifts. For the remaining 35 patients, we were able to calculate chronotype (MSF_sc_) both before and after stroke (Figure 1). Baseline characteristics of the 35 patients are provided in Tables 1 and 2. Strokes occurred between July 2011 and January 2012. Patients filled out the study questionnaires on average 68 ± 24 (SD) days post stroke. Mean NIHSS at hospital admission was 4.4 ± 4.2 and mean mRS was 2.3 ± 1.5. NIHSS and mRS were significantly reduced at discharge compared to hospital admission (Table 2). Fifteen of the 35 participants received fibrinolytic agents. We did not find significant differences between the 197 initially contacted patients and the final 35 participants regarding age, sex, time points of stroke, and stroke severity (data not shown). Stroke location was only available for the final participants.

**Table 1:** **Characteristics of all 35 stroke patients at time point of study participation/hospital admission**.

ID	Variable													
	Sex	Age	MSF_sc_		Work		NIHSS		mRS		Localization	Month	Full hour	Dem./Depr
			1	2	1	2	1	2	1	2				
2	F	57	3.2	3.2	Y	Y	1	1	1	1	MCA l	July	20:00	No/no
3	F	69	4.2	4.0	N	N	1	1	1	1	MCA r	July	20:00	No/no
4^a^	M	68	3.0	3.0	N	N	4	0	3	0	MCA l, PCA l	July	07:00	No/no
5	M	59	2.4	2.4	Y	Y	1	0	1	0	MCA r	August	20:00	No/no
7	M	58	2.1	2.5	Y	Y	5	1	2	1	MCA l	July	14:00	No/no
8	M	53	4.0	3.6	Y	Y	2	0	1	0	MCA l	August	07:00	No/no
10	M	87	2.3	1.1	Y	N	6	2	3	2	MCA l	July	13:00	Yes/no
11	F	79	3.5	4.0	Y	Y	0	0	0	0	MCA r	September	22:00	No/no
12^a^	M	63	3.6	2.2	Y	N	9	14	5	5	MCA r	July	07:00	No/no
15^a^	M	62	2.4	2.6	Y	Y	8	0	5	0	Ventrobasilar	July	16:00	No/no
17^a^	M	72	2.6	2.8	N	N	1	1	1	1	Ventrobasilar	August	14:00	No/no
18	F	82	4.3	4.3	N	N	2	2	1	1	Ventrobasilar	August	17:00	No/no
19	M	54	3.3	3.3	Y	Y	1	1	1	1	MCA l	July	05:00	No/no
20	F	77	3.3	2.8	Y	Y	6	2	3	1	MCA bs	October	04:00	No/no
21^a^	M	66	4.0	4.1	N	N	16	0	5	0	MCA r	October	17:00	No/no
22^a^	F	49	4.2	3.3	Y	N	3	2	2	2	MCA l	September	21:00	No/no
23	M	65	1.8	1.8	Y	Y	1	1	1	1	MCA r	October	05:00	No/no
25	F	87	3.8	3.8	Y	Y	0	0	0	0	MCA r, PCA r	October	20:00	No/no
26^a^	F	27	4.7	3.5	Y	N	6	4	2	2	PCA l	October	04:00	No/no
27^a^	M	37	4.2	4.2	Y	Y	1	0	1	0	Ventrobasilar	September	20:00	No/no
30^a^	M	56	2.8	3.5	Y	Y	10	2	4	1	Ventrobasilar	November	23:00	No/no
32	M	48	3.8	2.0	Y	n/a	6	3	2	1	MCA l	November	15:00	No/no
33^a^	F	62	4.4	3.8	Y	N	5	0	1	0	MCA l	October	03:00	No/no
34^a^	F	78	3.2	1.4	Y	N	17	15	5	5	MCA l	October	10:00	No/yes
35^a^	M	65	2.9	3.0	Y	N	8	3	4	2	AChA r	November	13:00	No/yes
36	M	89	2.8	1.5	N	N	11	6	4	3	MCA r, PCA r	November	07:00	No/no
37^a^	M	67	3.3	3.3	N	N	2	0	1	0	MCA l, PCA l	November	10:00	No/no
39	M	76	2.8	2.2	Y	Y	4	2	2	2	MCA l	December	10:00	No/no
42	F	69	3.8	3.8	Y	Y	2	2	2	2	AChA l	January	15:00	No/no
43^a^	M	62	2.4	2.4	Y	Y	4	0	4	0	Vertebrobasilar	January	21:00	No/no
44	M	73	3.5	3.5	N	N	1	0	1	0	MCA l, PCA	January	12:00	No/no
45^a^	F	81	5.0	3.0	N	N	3	0	3	1	MCA l	January	17:00	No/no
48	M	63	2.1	2.1	N	N	5	1	2	1	AChA l	January	06:00	No/no
49	M	73	3.8	3.7	N	N	1	1	1	1	MCA r	December	21:00	No/no
50^a^	F	86	3.6	3.6	Y	Y	2	0	4	1	PCA l	December	19:00	No/no

*Sex *f*emale, *m*ale/MSFsc (1) chronotype before (2) after stroke/Work (1) employed before (2) after stroke, *y*es, *n*o/Localization = stroke localization with MCA, middle cerebral artery, PCA, posterior cerebral artery, AChA, anterior choroidal artery with *l*eft, *r*ight, *bs* = both sides/*Month* of stroke/NIHSS/mRS (1) at hospital admission (2) at discharge/*Dem*entia/*Depr*ession*.

*^a^patient received a fibrinolytic agent*.

**Table 2:** **Characteristics of 35 (13 female) stroke patients at admission to and discharge from hospital**.

Variables	At admission		At discharge		*p*-Value
	Mean	SD	Mean	SD	
Age (years)	66.3	14.0	–	–	–
Stroke time point (h)	13.6	6.2	–	–	
NIHSS	4.4	4.2	1.9	3.4	<0.001^a^
mRS	2.3	1.5	1.1	1.3	<0.001^a^

*^a^Wilcoxon signed ranks test*.

**Figure 2:**
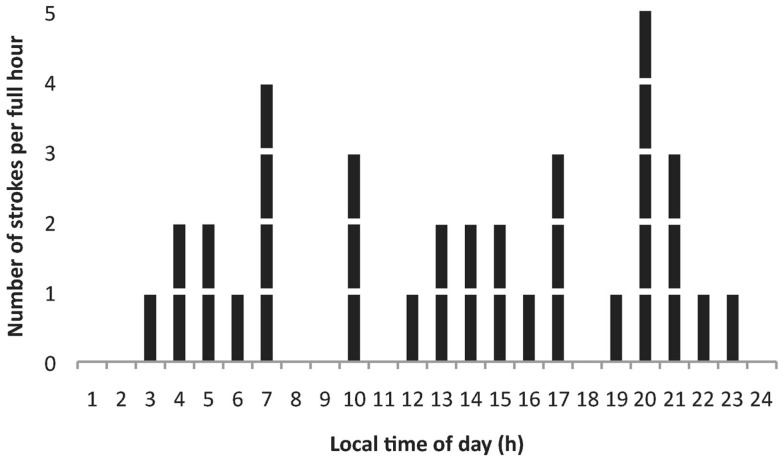
**Distribution of the number of strokes by time of day in 35 (13 female) stroke patients**. Most strokes per hour were observed at 07:00 and 20:00 h.

Figure 2 shows the distribution of strokes by time of day in the 35 patients. Sixty percent of strokes occurred between noon and midnight. The highest number of strokes was found at 8 p.m. (14%) and the second highest number at 7 a.m. (11%). Twenty-six percent were classified as wake up strokes.

Comparing the MCTQ entries from before and after stroke revealed significantly longer sleep durations on workdays and work-free days after stroke (Table 3). Chronotype (MSF_sc_) changed significantly after stroke, whereas social jetlag remained statistically unchanged (Table 3).

**Table 3:** **Sleep timing from MCTQ in 35 (13 female) patients before and after stroke**.

	Before stroke		After stroke		Difference	*p*-Value
	Mean	SD	Mean	SD
Sleep latency on workdays (min)	19.0	24.3	17.0	17.5	−2.0	0.552^§^
Sleep onset on workdays (h)	−0.7	0.9	−1.2	1.3	−**0.5**	0.011*
Sleep end on workdays (h)	6.6	1.2	6.8	1.2	−0.2	0.197*
Sleep inertia on workdays (min)	13.6	19.7	18.4	27.4	+4.8	0.106^§^
Sleep duration on workdays (h)	7.3	1.0	8.0	1.6	**+0.7**	0.010*
Sleep latency on free days (min)	19.6	24.3	15.4	16.1	−4.2	0.484^§^
Sleep onset on free days (h)	−0.4	0.9	−1.0	1.3	−**0.6**	0.002*
Sleep end on free days (h)	7.4	0.9	7.3	1.0	−0.1	0.558*
Sleep inertia on free days (min)	18.8	20.3	20.2	27.8	+1.4	0.675^§^
Sleep duration on free days (h)	7.7	1.1	8.3	1.5	**+0.6**	0.023*
Chronotype (MSFsc, h)	3.3	0.8	3.0	0.8	−**0.3**	0.014*
Social jetlag (h)	0.6	0.8	0.4	0.6	−0.2	0.108^§^

Bold numbers indicate statistically significant differences with *paired *t*-test and ^§^Wilcoxon signed ranks test.

The time point of stroke was not associated with a patients’ MSF_sc-before-stroke_ (*R* = 0.092, *p* = 0.607; Spearman correlation). Since MSF_sc_ was found to be on average significantly advanced after stroke, we calculated the individual MSF_sc_ differences (MSF_sc-after-stroke_ minus MSF_sc-before-stroke_) and correlated this individual MSF_sc_ difference with stroke severity at both hospital admission and discharge. There was a significant negative correlation between NIHSS and mRS at discharge with the difference in chronotype after stroke (Table 4), which remained significant after controlling for the number of days between stroke and study participation (*r* = −0.565, *p* = 0.001 for NIHSS at discharge, and *r* = −0.620, *p* < 0.001 for mRS at discharge; Spearman correlation). These correlations indicate that patients with more severe strokes showed more pronounced changes in their sleep timing.

**Table 4:** **Significant correlations between MCTQ entries and stroke time point with NIHSS and mRS in 35 (13 female) patients**.

Variables	Admission				Discharge			
	NIHSS		mRS		NIHSS		mRS	
	*R*	*p*	*R*	*p*	*R*	*p*	*R*	*p*
Δ MSFsc (h)	−0.24	0.180	−0.16	0.377	−0.38	0.031*	−0.49	0.004*
Δ Sleep onset workdays (h)	−0.45	0.006*	−0.37	0.029*	−0.39	0.020*	−0.42	0.013*

Δ = difference in entries to the MCTQ between before and after the stroke/*Spearman correlation.

**Figure 3:**
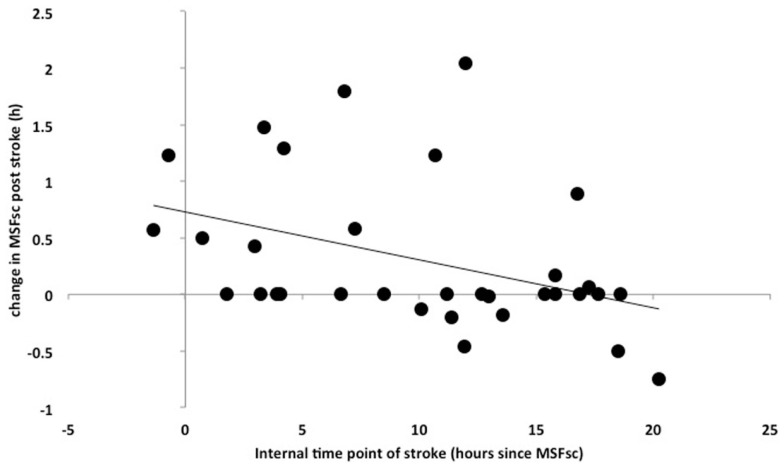
**Correlation between Int_stroke_ (MSF_sc-before-stroke_ − local time point of stroke) (*x*-axis) and the difference in MSF_sc_ after stroke (MSF_sc-after stroke_ − MSF_sc-before stroke_) (*y*-axis) in 35 stroke patients (13 female) (Spearman correlation, *R* = 0.421, *p* = 0.015)**. The correlation shows that the closer the time point of a stroke is to an individual’s MSF_sc_, the larger the advance (expressed as positive values on the *y*-axis) in chronotype (MSF_sc_) after the stroke.

**Figure 4:**
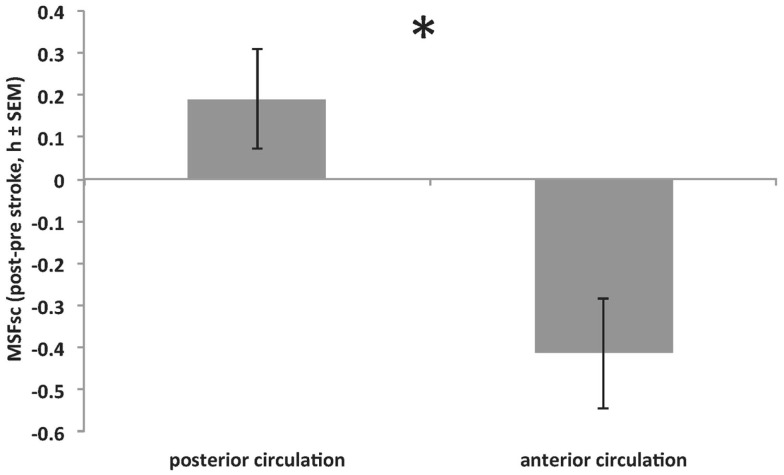
Significant difference in MSF_sc_ changes (MSF_sc-after stroke_ − MSF_sc-before stroke_) between strokes within the anterior circulation (middle, anterior and posterior cerebral artery and anterior choroidal artery; providing blood to the cerebrum) and strokes within the posterior circulation (ventrobasilar and vertebrobasilar artery; proving blood to the cerebellum and brain stem) (*p = 0.016, Mann–Whitney *U* test). Positive values on *y*-axis indicate MSF_sc_ advances. See also Table 1 for stroke locations.

Int_stroke_ was assessed to explore associations between the internal time point of stroke (relative to an individual’s MSF_sc_) and stroke severity. The closer a stroke happened to a patient’s MSF_sc-before stroke_, the larger the advance of MSF_sc_ at 3 months after stroke (Figure 3). This association remained significant after using the number of days between stroke and study participation as covariate (*R* = 0.398, *p* = 0.024; Spearman correlation). In addition, there was a significant difference in MSF_sc_ changes between stroke locations. Strokes that occurred in the anterior circulation lead to delays of MSF_sc_, whereas strokes within the posterior circulation lead to advances of MSF_sc_ (Figure 4).

The standard clinical assessment at hospital admission showed a number of risk factors and co-morbidities that were evenly distributed among the participants, including arterial hypertension, atrial fibrillation, rheumatoid arthritis, back problems, hypercholesteremia, smoking, and diabetes mellitus Type II. Medication at discharge did not substantially vary among participants. Most common drugs were acetylsalicylic acid, simvastatin, and ramipril (Table 5).

**Table 5:** **Medication at hospital discharge in all 35 (13 female) stroke patients**.

ID	Medication name
2	Pantoprazole, dronedarone, phenprocoumon, simvastatin
3	Ramipril, clopidogrel, amlodipine, indacaterol, beclometasone dipropionate, tiotropium bromide, atorvastatin, indapamide
4	Aspirin, dipyridamole, telmisartan, pantoprazole, allopurinol, metoprolol succinate, fluvastatin, alfacalcidol
5	Acetylsalicylic acid
7	Clopidogrel, acetylsalicylic acid, amlodipine, bisoprolol, atorvastatin
8	Acetylsalicylic acid, simvastatin
10	Acetylsalicylic acid, simvastatin, bisoprolol, enalapril, hydrochlorothiazide, allopurinol, levothyroxine, phenprocoumon, tiotropium bromide, formoterol
11	Acetylsalicylic acid, simvastatin, cortison, calcium, vitamin D, indapamide
12	Metoprolol, acetylsalicylic acid, simvastatin, dalteparin
15	Ramipril, amlodipine, doxazosin, acetylsalicylic acid, potassium citrate
17	Clopidogrel, lercanidipine, simvastatin, levothyroxine, lisinopril, hydrochlorothiazide, allopurinol
18	Acetylsalicylic acid, simvastatin, latanoprost eye drops, bisoprolol
19	Acetylsalicylic acid, simvastatin, ramipril
20	Acetylsalicylic acid, levothyroxine, roxythromycin, lorsartan, metoprolol succinate, spironolacton, hydrochlorothiazide, torasemide
21	Simvastatin, acetylsalicylic acid, glimepiride, metformin, verapamil, phenprocoumon
22	Simvastatin, acetylsalicylic acid
23	Acetylsalicylic acid, ramipril, metformin, pantoprazole, simvastatin
25	Acetylsalicylic acid, simvastatin
26	Acetylsalicylic acid
27	Acetylsalicylic acid
30	Acetylsalicylic acid, simvastatin, citalopram
32	Acetylsalicylic acid, simvastatin
33	Acetylsalicylic acid, simvastatin, ramipril, pantoprazole, diclofenac
34	Ramipril, simvastatin, bisoprolol, dabigatran, fluoxetine
35	Acetylsalicylic acid, simvastatin, ramipril, amlodipine, metoprolol succinate, citalopram
36	Acetylsalicylic acid, fraxiparin, marcumar, pravastatin, prednisolon, molsidomin, carvedilol, ramipril, kalinor, pantozol, tamsulosin, cefuroxim
37	Acetylsalicylic acid, metroprolol, amlodipin, brenzbromaron
39	Enalapril, marcumar, acetylsalicylic acid, fragmin P, cordarex, sortis
42	Acetylsalicylic acid, simvastatin, candesartan, timolol at
43	Acetylsalicylic acid, simvastatin
44	Acetylsalicylic acid, dabigatran, carmen, HCT, metoprolol, pantozol, simvastatin
45	Acetylsalicylic acid, l-thyroxin, simvastatin
48	Clopidogrel, metoprolol, simvastatin, ramipril
49	Acetylsalicylic acid, simvastatin
50	Levocomp, symbicort 1 hub, bisoprolol, pantozol, novamin, acetylsalicylic acid

## Discussion

Chronotype (MSF_sc_) and sleep duration on both workdays and work-free days changed significantly after stroke in our group of patients. The change in MSF_sc_ after stroke was negatively correlated with stroke severity (NIHSS and mRS at hospital discharge), which was independent of the number of days between stroke and study participation [on average 68 ± 24 (SD) days]. More pronounced motor deficits in these patients leading to also less time outdoors in sunlight could explain this finding, and physical activity has been shown to impact the circadian rest-activity system (28). We did not observe significant associations between month of stroke and MSF_sc_ changes and, therefore, exclude an effect of seasonal changes in light exposure on the observed chronotype differences. Twenty percent of our patients reported to not continue working after stroke, which could have impacted their sleep timing. However, patients who did not continue working after stroke still indicated different sleep times on workdays and work-free days in their MCTQ after stroke. This finding suggests that even in the absence of job obligations, these patients kept their daily routines similar to as they did before their stroke (e.g., due to early morning rehabilitation appointments after stroke compared to early morning work schedules before stroke). We did not find significant differences in social jetlag (a marker for displaced sleep because of early morning obligations) at 3 months after stroke, suggesting negligible impact on sleep from changes in working status.

Animal studies have demonstrated adverse effects of stroke on sleep architecture (6, 7, 9) and experimentally induced stroke in animal models has been shown to destabilize circadian rhythms as evidenced by altered timing of melatonin secretion (8). Thus, acute lesions in the central nervous system after stroke might result in circadian phase shifts. Changes in sleep homeostasis with prolonged sleep latencies, fragmented sleep pattern, and disturbed NREM sleep were shown in human stroke patients (10, 11) and discussed in the context of reduced subjective quality of life (29). Here, we did not find significant changes in sleep latency in our participants. Only few studies addressed circadian phase shifts in human stroke patients, by assessing changes in urinary melatonin secretion after stroke (12, 13). No studies in humans have assessed differences in phase of entrainment as a result of stroke as we did in the current study with the MCTQ.

Concerning stroke onset in relation to time of day, our finding of an evening peak at 8 p.m., and a second peak at 7 a.m. is in line with previous reports (2–5). The bimodality in the distribution of stroke onsets across the 24-h day indicates the potential for chronotherapies tailored for more individualized medicine, as also elaborated on previously by Manfredini and co-workers (3).

We found no correlation between MSF_sc_ (chronotype) and stroke onset. Rather, the impact of stroke on MSF_sc_ varied with the internal time of stroke (InT_stroke_). This finding was independent of the number of days between stroke onset and study participation – the closer a stroke happened to an individual’s MSF_sc-before-stroke_, the stronger the advance of MSF_sc_ at the time of follow-up.

In addition, we found that strokes within the anterior circulation affected MSF_sc_ significantly different compared to strokes within the posterior circulation. Because of our small sample size, we can only speculate on the implications of this finding. For example, the anterior circulation supplies blood to the cerebrum, whereas the posterior circulation provides blood to the cerebellum and brain stem. Since the ascending arousal system (AAS) of the brain stem (30) regulates the transitions between sleep and wake, a stroke in the AAS, therefore, might disturb the sleep/wake switch (31) leading to changes in sleep timing. In addition, reduced blood flow to the cerebellum could lead to impaired locomotor activity, which, in turn, might lead to earlier sleep times. In contrast, the anterior circulation also provides blood to the suprachiasmatic nucleus (SCN), which is key to the circadian entrainment process (32, 33). Future studies using, for example, brain scan techniques will help to verify if temporally reduced blood and glucose supply to SCN neurons impacts circadian entrainment properties in stroke patients. In addition, the SCN is intimately connected within the central nervous system via a host of afferent and efferent projections (34, 35). Investigations of neurological deficits after stroke related to compromised functions of brain structures upstream and/or downstream of the SCN are highly warranted. The current study found delays of MSF_sc_ in patients with strokes that occurred early within their internal day, whereas MSF_sc_ rather advanced after strokes occurring at later internal time points. Therefore, one could hypothesize that stroke vulnerability within blood circuits (e.g., depending on blood vessel type or size) might vary with internal time. A time-of-day effect, for example, of stroke severity has been shown, with strokes happening during sleep being more severe than strokes happening during wakefulness (36).

Strength of our study is a homogenous sample of only first-ever ischemic stroke patients. As we did not find statistically significant differences in baseline characteristics such as age, sex, time point of stroke, and stroke severity between study participants and the initially contacted patients, we consider our study sample to be representative for the initial population. Limitations of our study are the retrospective study design and a participation rate of 28%, which was not caused by a lower stroke severity in participants compared to non-participants. A response rate of about 30% is, however, to our knowledge not unusual for cross-sectional assessments by mail in clinical populations. In addition, because 25% of the 56 initial participants expressed interest in an individual sleep evaluation, we cannot exclude that this incentive to participate in the study caused a selection bias in our study population. Two of the 35 participants showed signs of depression at hospital admission (IDs 34 and 35, Table 1), and 1 patient was diagnosed with signs of dementia (ID 10, Table 1), which renders these factors unlikely to influence the main outcome. However, we did not have information about the clinical condition (e.g., depression status, prevalence of sleep problems) of the patients at the time of the survey, which might have influenced our findings, since depression after stroke has been shown to predict sleep disturbances (37). Since it is impossible to know the occurrence of a stroke beforehand, prospective studies following people at high risk for vascular events should include both assessments of external (local) and internal (biological) time. *Post hoc* power and sample size calculations performed using SAS JMP 7.0 software showed a statistical power of 58% of the current data set with 58 participants needed to obtain a statistical power of 80%.

In conclusion, this is the first study that provides evidence for changes in chronotype (MSF_sc_) at up to 3 months after stroke, which were associated with both stroke severity and the internal time point at which a stroke occurred. We did not find a significant association between stroke onset and MSF_sc_. Future studies are needed to scrutinize our findings in larger samples with also applying daily sleep diaries and objective measures (e.g., actigraphy or melatonin samples) to better quantify the circadian impact of stroke. We hope that our findings provide sufficient evidence to stimulate larger follow-up studies expanding to also other vascular and cardiac events like hemorrhagic strokes, heart attacks, atrial fibrillation, or pulmonary embolism.

## Conflict of Interest Statement

The authors declare that the research was conducted in the absence of any commercial or financial relationships that could be construed as a potential conflict of interest.
